# First mitochondrial genome for the red crab (*Charybdis feriata*) with implication of phylogenomics and population genetics

**DOI:** 10.1038/srep11524

**Published:** 2015-07-30

**Authors:** Hongyu Ma, Chunyan Ma, Chenhong Li, Jianxue Lu, Xiong Zou, Yangyang Gong, Wei Wang, Wei Chen, Lingbo Ma, Lianjun Xia

**Affiliations:** 1 East China Sea Fisheries Research Institute, Chinese Academy of Fishery Sciences, Shanghai 200090, China; 2 Key Laboratory of East China Sea and Oceanic Fishery Resources Exploitation, Ministry of Agriculture, Shanghai 200090, China; 3 College of Fisheries and Life Science, Shanghai Ocean University, Shanghai 201306, China

## Abstract

In this study, we first described the complete mitochondrial genome for the red crab (*Charybdis feriata*), elucidated its phylogenetic relationship among 20 species within Decapoda, and estimated the population genetic diversity. The mitochondrial genome was 15,660 bp in size and encoded 13 protein-coding genes, 22 transfer RNA (tRNA) genes, and two ribosomal RNA genes. The gene arrangement of the mitochondrial genome was the same as that of its sister species, *C. japonica*. Phylogenomic analysis suggested that genus *Charybdis* should be classified into subfamily Portuninae but not into subfamily Thalamitinae. Moreover, a total of 33 haplotypes of complete cytochrome *c* oxidase subunit I gene were defined in 70 individuals of *C. feriata* derived from three localities. Haplotype diversity and nucleotide diversity values among three localities indicated a high level of genetic diversity in *C. feriata*. AMOVA analysis suggested a low level of genetic differentiation among the three localities (*F*_ST_ = 0.0023, *P* > 0.05). Neutrality tests and mismatch analysis revealed that *C. feriata* might have undergone a population expansion event that possibly occurred in the last 61,498 to 43,814 years. This study should be helpful to better understand the evolutionary status, and population genetic diversity of *C. feriata* and related species.

The red crab, *Charybdis feriata* (Crustacea: Decapoda: Portunidae) (Linnaeus, 1758), also known as the crucifix crab, is a large swimming crab species that is broadly distributed in the Indo-Pacific sea areas, including Japan, China, Indonesia, Australia, India, Pakistan, Oman, and South Africa^1^,^2^. *C. feriata* can be easily distinguished by its striking red and white colour pattern and the distinct cross on the median surface of its carapace^3^. Young crabs usually live in the sandy shore, whereas adults prefer to inhabit the areas of sandy and muddy bottoms at depths from 30 m to 60 m^4^. Given its fast growth speed, large size, good flavor, and high market demand, this species is considered as one of most valuable fisheries resources and has become a potentially important target for aquaculture, domestication, and stock enhancement^5^,^6^. The wild females usually weigh 200 - 350 g, but the males can grow up to 1 kg^7^. In the last few decades, the catching production and the wild resources of *C. feriata* have been decreasing on a yearly basis^8^ due to over-exploitation and environmental deterioration. Despite its economic importance, studies have been limited to reproductive biology^7^,^8^, larvae characteristics^9^,^10^, and fishery biology^11^. Little information could be available about the germplasm resource and population genetic structure for this species by now, except that moderate variation was reported for two wild populations sampled from Shanwei City and Zhoushan City, China based on the cytochrome *c* oxidase subunit I (COI) gene and microsatellite markers, respectively^12^,^13^. To estimate and conserve this important crab resource, genetic studies such as population genetic diversity and evolutionary history should be carried out.

The genus *Charybdis* (De Haan, 1833) is an important group in family Portunidae. For a long time, the taxonomic status of this genus has remained controversial. Several studies have recommended to classify it into subfamily Portuninae of family Portunidae^14^,^15^,^16^. However, some other studies suggested to assign it to subfamily Thalamitinae of family Portunidae^17^,^18^. Molecular studies based on COI, 16S rRNA, and RAPD^19^,^20^ supported the latter opinion. However, the phylogenetic analysis based on 13 protein-coding genes from the mitochondrial genome^21^ supported the former opinion. Thus, in order to better solve this problem, more studies need to be carried out in the future.

Mitochondrial genome is a typically closed-circular molecule ranging approximately from 14 to 18 kb in size, and it consists of 13 protein-coding genes, 22 transfer RNA (tRNA) genes, 2 rRNA genes, and a control region. It is thought to be an ideal marker for studies on population genetic diversity, molecular phylogeny, and species identification because of its high mutation rate, simple structure, abundant distribution, and maternal inheritance^22^,^23^,^24^. Thus far, complete mitochondrial genomes have been reported in many crustacean species, such as *Litopenaeus vannamei*^25^, *C. japonica*^21^, *Scylla serrata*^26^, and *S. paramamosain*^23^. Although several mitochondrial gene sequences of *C. feriata* are present in the GenBank database, the complete mitochondrial genome information is still not available by now. The lack of complete mitochondrial genome has limited the development of population genetic diversity and molecular evolution for this species.

The purpose of this study is to report the complete mitochondrial genome for *C. feriata*, elucidate its evolutionary status, and estimate population genetic diversity and differentiation. This work should be helpful to better understand the evolutionary status and population genetic diversity of *C. feriata* and other related crustacean species.

## Materials and methods

### Sampling and DNA extraction

**Figure 1:**
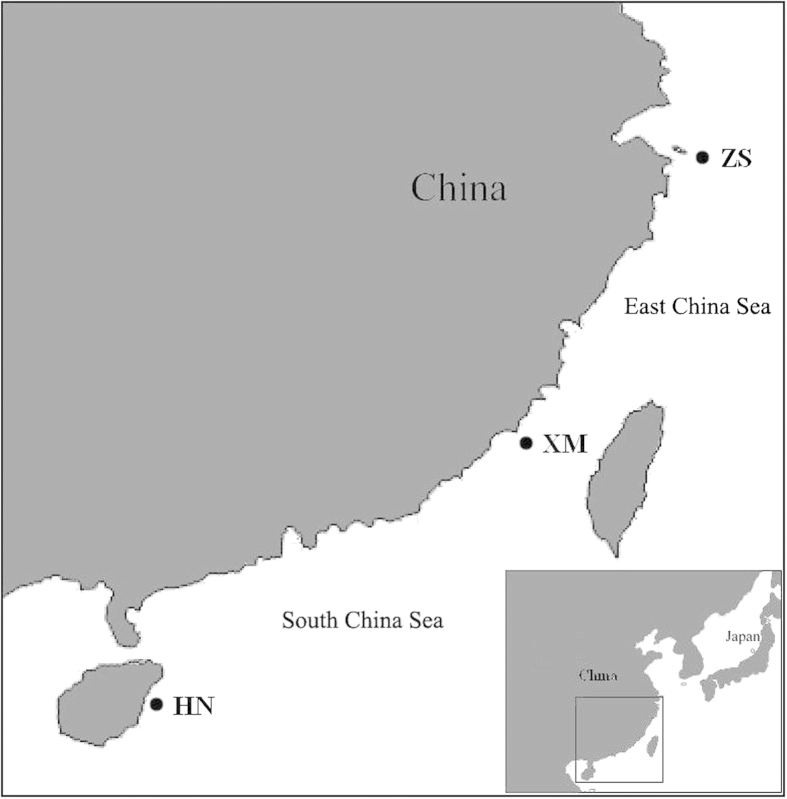
Three localities of *Charybdis feriata* collected in this study. HN, Hainan locality; XM, Xiamen locality; ZS, Zhoushan locality. This map was created by the software Adobe Photoshop 7.0.

A total of 70 wild individuals of *C. feriata* were sampled from the southeastern coasts of China, with 21 from Hainan Island (named HN), 24 from the city of Xiamen (named XM), and 25 from the city of Zhoushan (named ZS) (Fig. 1). Animals were killed by a lethal dose of MS-222. Muscle tissues were collected and fixed in 99% ethanol at room temperature. Genomic DNA was extracted using the traditional proteinase K and phenol-chloroform extraction protocol as described by Ma^27^.

### Primers, PCR, and sequencing

First, partial sequences of three genes (12 S rRNA, 16 S rRNA, and COI) of *C. feriata* and the complete mitochondrial genomes of three closely related crab species (*C. japonica, S. paramamosain*, and *Portunus trituberculatus*) were downloaded from the GenBank database. Then the three genes were confirmed by resequencing. Next, the complete mitochondrial genome of *C. feriata* was generated by overlapping PCR with specific or degenerate primers (Supplementary Table 1), and sequencing. Furthermore, the complete COI gene sequence was employed to evaluate the population genetic diversity and genetic differentiation of *C. feriata* population. A pair of primers (COI-f: 5′–AATAAGAAAGTTAATAACTTGTGTT–3′ and COI-r 5′–GAAGAAAAGTATCTTCCTAGTAGG–3′) with an anealing temperature of 52 °C were successfully designed. Seventy individuals collected from three localities (HN, XM, and ZS) were evaluated in this study.

PCRs were carried out in a 25 μL volume that included 0.4 μM each primer, 0.2 mM each dNTP, 1 × PCR buffer, 1.5 mM MgCl_2_, 0.75 unit *Taq* polymerase, and approximately 100 ng template DNA at the following conditions: one cycle of denaturation at 94 °C for 4 min; 37 cycles of 30 s at 94 °C, 50 s at a primer-specific annealing temperature (Supplementary Table 1), and 50 s at 72 °C. As a final step, the products were extended for 7 min at 72 °C. The PCR products were separated on 1.5% agarose gels and directly sequenced in both directions by using an ABI Prism 3730 automated DNA sequencer (PE Corporation). The sequences were edited and assembled using two softwares, EditSeq and SeqMan (DNASTAR).

### Complete mitochondrial genome analysis

**Figure 2:**
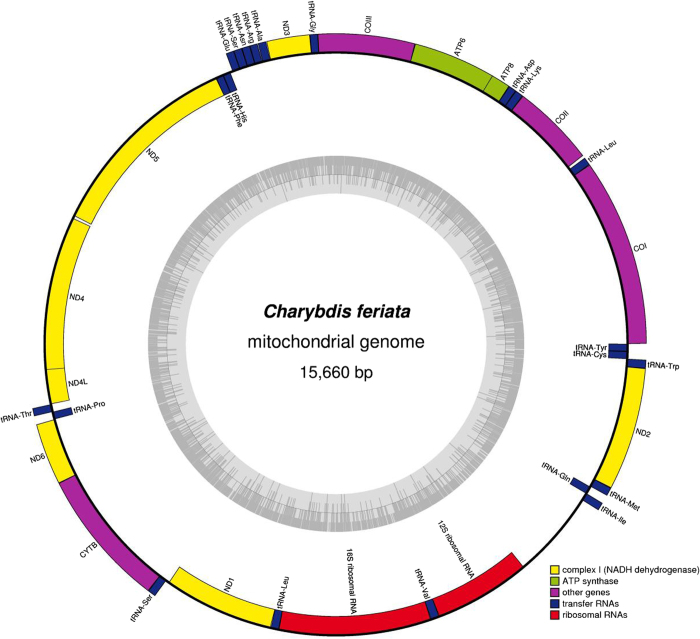
Graphical map of complete mitochondrial genome of *Charybdis feriata*. Genes encoded by the heavy strand were shown outside the circle, and encoded by the light strand were shown inside the circle respectively. The inner ring showed the GC content of this genome.

The graphical map of the complete mitochondrial genome (Fig. 2) was drawn using the online software OrganellarGenomeDRAW (http://ogdraw.mpimp-golm.mpg.de/)^28^. The genome structure was determined by sequence comparisons with the known complete mitochondrial genomes of the closely related species, including *S. paramamosain*^23^ and *C. japonica*^21^. tRNAs were identified by their proposed clover-leaf secondary structure and anticodons by using the web-based tRNA-scan SE 1.21 program (http://lowelab.ucsc.edu/tRNAscan-SE/)^29^ with default search mode. Protein-coding genes were translated into amino acids by using the software MEGA 4.0^30^. The codon usage of protein-coding genes and the nucleotide composition of the mitochondrial genome were also determined using MEGA 4.0. Finally, the complete mitochondrial genome DNA sequence was deposited into the GenBank database by using the software Sequin 12.30 (http://www.ncbi.nlm.nih.gov/Sequin/).

### Phylogenomic analysis

The complete mitochondrial genomes of 19 species under Decapoda were downloaded from the GenBank database, including *Charybdis japonica* (FJ460517), *Callinectes sapidus* (NC_006281), *Eriocheir japonica* (NC_011597), *Eriocheir hepuensis* (NC_011598), *Eriocheir sinensis* (NC_006992), *Geothelphusa dehaani* (NC_007379), *Fenneropenaeus chinensis* (DQ518969), *Litopenaeus vannamei* (DQ534543), *Pagurus longicarpus* (NC_003058), *Macrobrachium rosenbergii* (NC_006880), *Marsupenaeus japonicus* (NC_007010), *Penaeus monodon* (NC_002184), *Portunus trituberculatus* (AB093006), *Panulirus japonicus* (NC_004251), *Pseudocarcinus gigas* (NC_006891), *Scylla serrata* (NC_012565), *Scylla tranquebarica* (NC_012567), *Scylla olivacea* (NC_012569), and *Scylla paramamosain* (JX457150). Furthermore, one species, *Harpiosquilla harpax* (NC_006916), was used as an outgroup taxon.

Protein-coding genes were aligned using Clustal W in MEGA 4.0 with default settings. As a result, gene ND6 showed high heterogeneity that consistently causes poor phylogenetic performance^31^. Thus, the remaining 12 protein-coding genes alignments were concatenated to a single multiple sequence alignment. Then the multiple sequence alignment was formatted and analyzed using RAxML web-servers (http://embnet.vital-it.ch/raxml-bb/index.php)^32^. The CAT model was used to estimate the evolutionary rate of the 12 protein-coding genes. Maximum likelihood (ML) search was carried out after bootstraps. The phylogenetic tree was drawn by the software FigTree v1.4.2.

### Population genetic analysis

Haplotypes were identified using software Dna SP version 5.0^33^ and deposited into the GenBank database. For each locality and overall locality, haplotype diversity (*h*) and nucleotide diversity (*π*) were calculated using Dna SP version 5.0. Molecular variance (AMOVA) analysis was carried out using software Arlequin version 3.11^34^ to explain the genetic structure and differentiation among these three localities. Significant level of the test was assessed using 1000 permutations of each pairwise comparison. Neutrality tests including Ewens-Watterson^35^,^36^, Chakraborty^37^, Tajima *D*^38^, and Fu’s *Fs*^39^ with 1000 permutations were performed using software Arlequin version 3.11. Mismatch analysis^40^ with 10000 bootstrap replicates were also performed using Arlequin 3.11. The histogram of mismatch distribution was constructed using the software Network version 4.6.1.2 (http://www.fluxus-engineering.com/)^41^. The median-joining network of haplotypes was also constructed using software Network version 4.6.1.2. The rough time of population expansion was estimated using the following equation *t* = *τ*/2*u*^42^, where *t* is the time since population expansion, *τ* is the mutational time scale, which is calculated using software Arlequin version 3.11, and 2*u* is calculated using the equation 2*u* = *μ* × length of sequence × generation time, where *μ* is the mutation rate. Given the lack of a calibrated mutation rate of COI gene of *C. feriata*, the mutation rates of COI gene ranging from 1.66% to 2.33% per million years of *Sesarma*^43^ were used. In addition, a generation time of one year was also used.

## Results and Discussion

### Genome organization

The mitochondrial genome of *C. feriata* was a typically circular molecule with 15,660 bp in size (GenBank accession no. KF386147) and consisted of 13 protein-coding genes, 22 tRNA genes, two rRNA genes, and a control region (Fig. 2) as found in most metazoan species such as *Lutra lutra*^44^, *C. Japonica*^21^, and *S. paramamosain*^23^. This genome was slightly smaller than those of most sequenced crab species under Decapoda but larger than that of *P. gigas*, whose size was 15,515 bp^21^,^23^,^26^,^45^. Such a small genome was mainly due to the short size of the control region (762 bp). Moreover, the lengths of other regions of the genome were approximately equal among these species. Heavy strand (H-strand) encoded 23 genes, whereas light strand (L-strand) encoded the remaining 14 genes. The arrangement and order (Table 1) of the 37 genes were completely identical to those of reported species of Portunids, such as *C. japonica*^21^ and *S. paramamosain*^23^. However, the location of tRNA^His^ (Table 1) was different from that of most arthropods. In most arthropods, tRNA^His^ was between NAD4 and NAD5, whereas it was found between tRNA^Glu^ and tRNA^Phe^ in *C. feriata*. The phenomenon of gene rearrangements in mitochondrial genome was a relatively common event in crustacean species^25^. The tandem duplication of gene regions was thought to be the most acceptable mechnism of mitochondrial gene rearrangement, in this case, the slipped-strand mispairing took place first, and then followed by gene deletions^46^.

Ten gene overlaps and ten intergenic spacers were found in mitochondrial genome of *C. feriata*, most of them have been reported in many other species mitochondrial genomes^23^,^24^,^47^. The total length of overlaps and intergenic spacers were 24 and 105 bp, with ranges from 1 to 7 and from 2 to 27 bp, respectively. The overall A + T content of *C. feriata* mitochondrial genome was 70.15%, which was similar to those of *C. japonica, S. olivacea, S. serrata*, and *P. trituberculatus* (Table 2). However, different regions had different A + T contents. The control region had the highest A + T content (78.74%), whereas the protein-coding region had the lowest A + T content (68.60%).

### Protein-coding genes

A total of 13 protein-coding genes were identified, of which 9 (COI, COII, COIII, APT6, ATP8, ND2, ND3, ND6, and Cyt *b*) were encoded by H-strand, and 4 (ND1, ND4, ND4L, and ND5) were encoded by L-strand. These genes consisted of 11,182 bp in length and coded 3716 amino acids in total. All 13 genes were initiated by the start codon ATN (ATG, ATA, and ATT), with an exception (GTG) in ATP8 (Table 1). The typical stop codon (TAA or TAG) was detected in nine protein-coding genes (ATP6, ATP8, ND1, ND2, ND3, ND4, ND4L, ND5, and ND6), whereas the remaining four genes (COI, COII, COIII, and Cyt *b*) were ended by incomplete stop codons (T-- or TA-). Variable start codons and incomplete stop codons have been reported in many other mitochondrial genomes. For example, four types of start codons (ATT, ATG, ATA, and ACG) were detected in mitochondrial genome of *Myrmeleon immanis*^48^. Two and one incomplete stop codons were found in *Lutjanus russellii* and *S. paramamosain* mitochondrial genomes^23^,^49^. For the incomplete stop codon, the missed nucleotides may be produced by post-transcriptional polyadenylation^50^. In addition, ND6 had the highest A + T content (72.78%), whereas COIII had the lowest A + T content (64.85%) (Table 3).

### Transfer and ribosomal RNA genes

**Figure 3:**
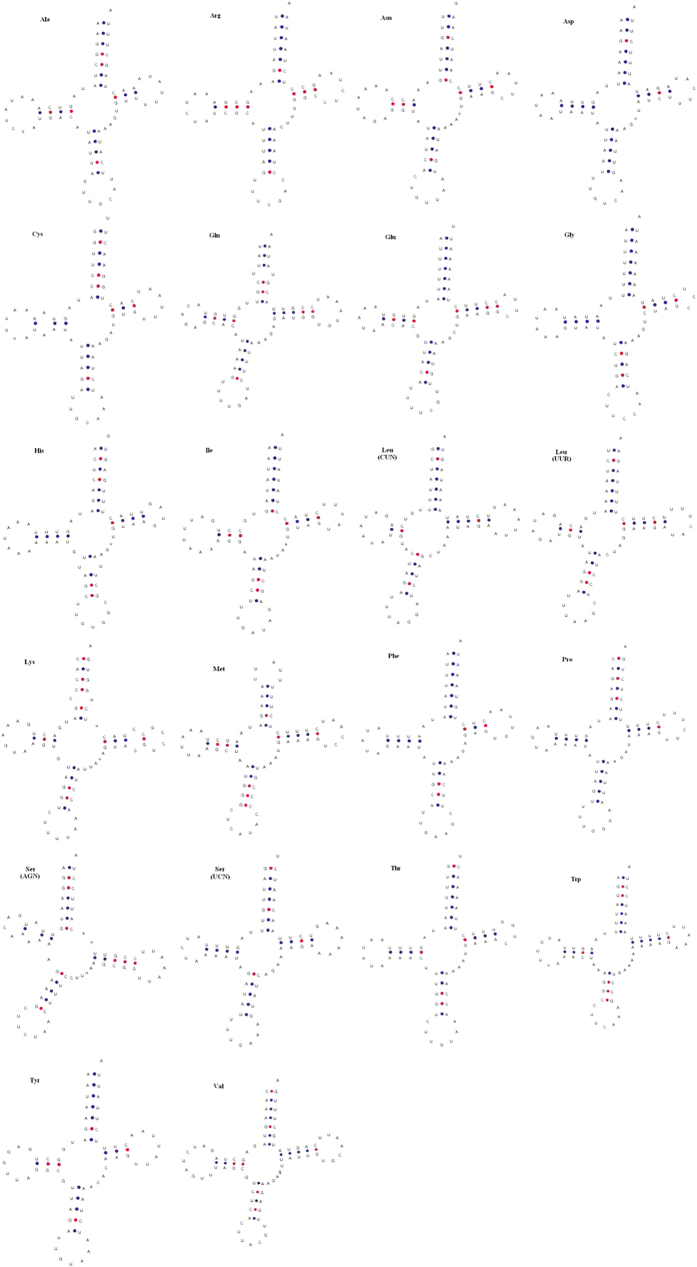
Putative secondary structures of 22 tRNAs detected from mitochondrial genome of *Charybdis feriata*.

A total of 22 tRNA genes ranging from 64 to 74 bp in length were identified from the mitochondrial genome of *C. feriata*. All of them were capable of folding into a typically clover-leaf secondary structure (Fig. 3). In the closely related crabs *C. japonica* and *S. paramamosain*, tRNA^Ser (AGN)^ could not form a secondary structure because it lacked the dihydrouracil (DHU) arms^21^,^23^. Fourteen tRNA genes were located on H-strand, whereas the remaining eight were located on L-strand. All tRNA genes had a common length of 7 bp for the aminoacyl stem and an invariable size of 7 bp for the anticodon loop. Variable nucleotide lengths of tRNAs were found at the DHU, T*Ψ*C, and anticodon arms. All these 22 tRNA genes possessed the common anticodons of Decapods mitochondrial genomes, except that tRNA^Lys^ and tRNA^Ser (AGN)^ possessed TTT and TCT anticodons rather than CTT and GCT, respectively. Seven unmatched base pairs were found in 22 tRNA genes, which was lower than the number detected from tRNA genes of *S. paramamosain*^23^. The overall A + T content of 22 tRNA was 71.76%, with the highest content (84.62%) in tRNA^Asp^ and the lowest content (57.35%) in tRNA^Lys^.

Both 16S and 12S rRNA genes were found on the L-strand of the mitochondrial genome. They were located between tRNA^Leu (CUN)^ and the putative control region and were separated by tRNA^Val^. The sizes of 16S rRNA and 12S rRNA genes were 1321 and 843 bp, and the A + T contents were 74.26% and 71.89%, respectively.

### Non-coding regions

A total of 11 non-coding regions were identified in the mitochondrial genome of *C. feriata*. The major non-coding region (762 bp in length) was found between 12S rRNA and tRNA^Ile^, which was considered to be the putative control region. The other 10 non-coding regions were small, ranging from 2 to 27 bp in length. The A + T content of control region was higher (78.74%) than that of other regions in mitochondrial genome. The high rate of A + T content was due to the existence of A/T repeated motifs. In control region, TA, AT, TAA, AAT, and TTA were found to be the most abundant motifs. Additionally, microsatellite sequences were detected, such as (TA)_3_, (AT)_3_, (TA)_4_, (AT)_4_, and (TA)_11_. Microsatellites were also identified from control region of the mitochondrial genome in *M. immanis* and *Nymphes myrmeleonoides*^48^. In addition, the nucleotide composition of control region in this study was 41.99% for A, 7.87% for G, 36.75% for T, and 13.39% for C (Table 3).

### Phylogenetic relationship

**Figure 4:**
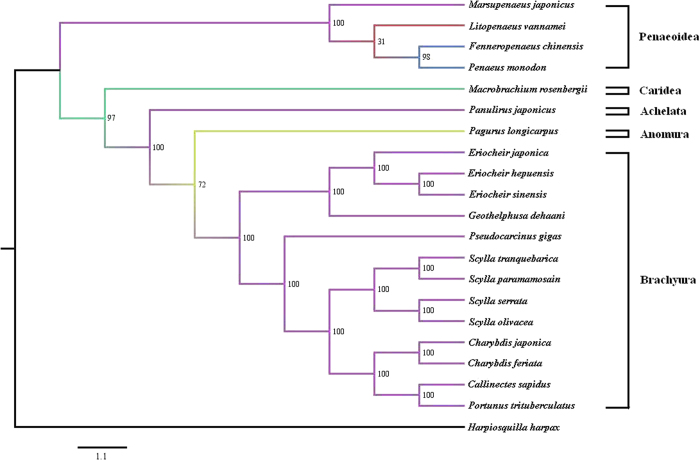
Phylogenetic relationship of Decapoda species based on 12 protein-coding genes.

The taxonomic status of genus *Charybdis* within Portunidae has been a highly contentious issue for a long time. In this study, we estimated the evolutionary relationship of *C. feriata* within Decapoda by reconstructing a phylogenetic tree. This tree was created based on 12 concatenated protein-coding genes from the mitochondrial genome of 21 species. From the tree topologies (Fig. 4), we found that *C. feriata* and *C. japonica* first formed a monophyletic group and showed the closest relationship to each other. Together with *C. sapidus* and *P. trituberculatus*, they then formed another monophyletic group. Furthermore, these four species showed a sister relationship with another four species of genus *Scylla*. Thus, our results supported the opinion of classifying genus *Charybdis* into subfamily Portuninae of family Portunidae. The same suggestion was also proposed by Liu and Cui^21^.

### Population genetic diversity and differentiation

The complete COI gene sequence (1534 bp) was employed to estimate the genetic diversity and differentiation of *C. feriata* population. A total of 33 haplotypes (Table 4) were identified from 70 individuals, of which 14 were from HN locality, 11 from XM locality, and 16 from ZS locality. H9 was the most abundant haplotype, which was present in each locality. A high level of genetic diversity (Table 5) was found with *h* ranging from 0.819 to 0.867 and *π* ranging from 0.0011 to 0.0013 per locality. The *h* value was slightly higher than that (0.787) reported by Huang^12^. In our previous study, moderate genetic variation of *C. feriata* was detected by microsatellite markers^13^. Moreover, a high genetic diversity was also observed in other marine animals, such as *S. serrata*^51^ and *Salmo salar*^52^. The following factors, including life history charateristics, environmental heterogeneity, and large population sizes, may help in maintaining a high level of genetic diversity among marine animals^53^,^54^. AMOVA analysis indicated that 99.77% of the total genetic variation was contributed by within-localities variation, whereas only 0.23% was caused by among-localities variation (*F*_ST_ = 0.0023, *P* > 0.05) (Table 6). In addition, no significant genetic differentiation was found among three localities (Table 7). The above analysis showed that the genetic differentiation among three localities of *C. feriata* was at a low level, suggesting a single population in East China Sea and South China Sea. The summer southwest monsoonal wind and winter northeast monsoonal wind drive seasonal ocean current circulation between South China Sea and East China Sea^55^,^56^. This homogenous population structure of *C. feriata* might be related with the ocean current circulation and the high larval dispersal ability of this crab species.

### Neutrality tests, mismatch analysis and population expansion estimation

**Figure 5:**
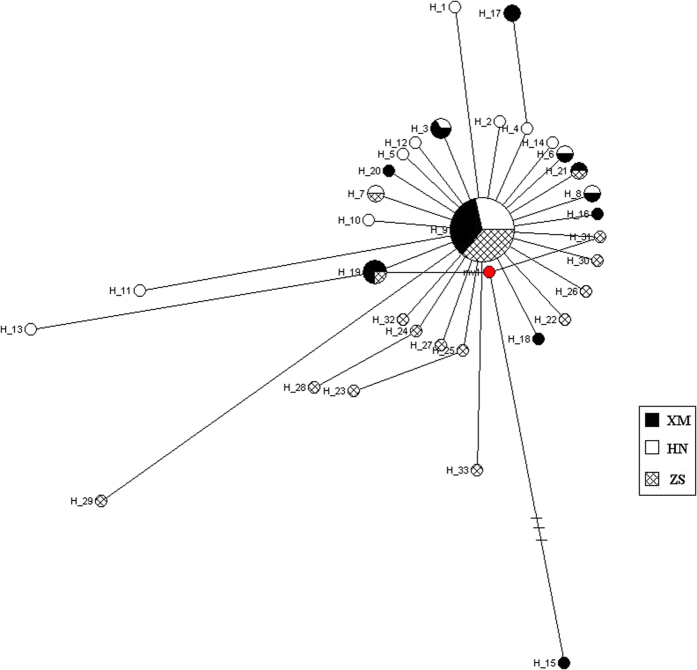
The median-joining network of 33 haplotypes of complete COI gene sequence of *Charybdis feriata.* XM, Xiamen locality; HN, Hainan locality; ZS, Zhoushan locality.

Four kinds of neutrality tests, including Ewens-Watterson, Chakraborty, Tajima *D*, and Fu’s *Fs*, were carried out in *C. feriata*. All of them, except Ewens-Watterson, suggested a significant deviation from mutation-drift equilibrium (Table 8). Mismatch distributions analysis (Table 9) indicated that the estimated effective population size after population growth was significantly larger than that before population growth. In addition, a star-like topology was produced based on 33 haplotypes (Fig. 5). Of these haplotypes, H9 was located in the center of this topology, and it was closely linked with the majority of other haplotypes, thereby suggesting that it is the ancestral haplotype in *C. feriata*. All above analysis indicated that *C. feriata* might have undergone a population expansion event. Meanwhile, the high *h* (between 0.819 and 0.867) and low *π* (between 0.0011 and 0.0013) also suggested that *C. feriata* underwent population expansion after a period of low effective population size. Sudden population expansion can affect population genetic diversity and haplotypes, and in this process more haplotypes were generated by mutation than were removed by genetic drift^57^. We further deduced that the population expansion event of *C. feriata* might have occurred between 61,498 and 43,814 years ago. This period of population expansion is a little bit later than the Last Interglacial complex (140-75 kya)^58^. This findings showed that the Last Interglacial complex might have played an important role in demographic history of *C. feriata*. Further, a big changes of nutrient concentrations and sea water temperature could affect the population distribution of marine orgnisms too^59^.

## Conclusion

This study first described the complete mitochondrial genome of *C. feriata*, which was 15,660 bp in length, including a typical set of 37 genes and a control region. Phylogenomic analysis results supported that genus *Charybdis* should be classified into subfamily Portuninae rather than into subfamily Thalamitinae. Furthermore, a high level of genetic diversity and a low level of differentiation of *C. feriata* were found, and a population expansion event was deduced to have occurred between 61,498 and 43,814 years ago. This study should be helpful for studies on evolution and phylogeny, population genetic structure, and conservaton genetics for *C. feriata* and related species^60^,^61^,^62^.

## Additional Information

**How to cite this article**: Ma, H. *et al.* First mitochondrial genome for the red crab (*Charybdis feriata*) with implication of phylogenomics and population genetics. *Sci. Rep.* **5**, 11524; doi: 10.1038/srep11524 (2015).

## Supplementary Material

Supplementary Information

## Figures and Tables

**Table 1:** Gene structure of mitochondrial genome of *Charybdis feriata*.

	**Position**			**Codon**				
**Gene**	**From**	**To**	**Size (bp)**	**Amino acid**	**Start**	**Stop**^a^	**Intergenic nucleotide (bp)**^b^	**Strand**^c^
COI	1	1534	1534	511	ATG	T--	0	H
tRNA^Leu (UUR)^	1535	1601	67				21	H
COII	1623	2307	685	228	ATG	T--	0	H
tRNA^Lys^	2308	2375	68				0	H
tRNA^Asp^	2376	2440	65				0	H
ATP8	2441	2602	162	53	GTG	TAG	−7	H
ATP6	2596	3273	678	225	ATT	TAA	−1	H
COIII	3273	4063	791	263	ATG	TA-	−1	H
tRNA^Gly^	4063	4126	64				0	H
ND3	4127	4480	354	117	ATT	TAA	3	H
tRNA^Ala^	4484	4550	67				3	H
tRNA^Arg^	4554	4618	65				0	H
tRNA^Asn^	4619	4685	67				0	H
tRNA^Ser (AGN)^	4686	4754	69				−1	H
tRNA^Glu^	4754	4819	66				22	H
tRNA^His^	4842	4907	66				0	L
tRNA^Phe^	4908	4971	64				−1	L
ND5	4971	6698	1728	575	ATG	TAA	20	L
ND4	6719	8053	1335	444	ATG	TAA	−7	L
ND4L	8047	8349	303	100	ATG	TAA	2	L
tRNA^Thr^	8352	8416	65				0	H
tRNA^Pro^	8417	8482	66				2	L
ND6	8485	8991	507	168	ATG	TAA	−1	H
Cyt*b*	8991	10125	1135	378	ATG	T--	0	H
tRNA^Ser(UCN)^	10126	10192	67				27	H
ND1	10220	11179	960	319	ATA	TAA	2	L
tRNA^Leu(CUN)^	11182	11250	69				0	L
16 S rRNA	11251	12571	1321				0	L
tRNA^Val^	12572	12645	74				0	L
12 S rRNA	12646	13488	843				0	L
Control region	13489	14250	762				0	
tRNA^Ile^	14251	14317	67				−3	H
tRNA^Gln^	14315	14383	69				3	L
tRNA^Met^	14387	14455	69				0	H
ND2	14456	15463	1008	335	ATG	TAA	−1	H
tRNA^Trp^	15463	15531	69				−1	H
tRNA^Cys^	15531	15595	65				0	L
tRNA^Tyr^	15596	15660	65				0	L

^a^, T-- or TA- represents incomplete stop codons.

^b^, Numbers correspond to the nucleotides separating adjacent genes. Negative numbers indicate overlapping nucleotides.

^c^, H or L indicates that the gene is encoded by the H or L strand.

**Table 2:** Comparison of mitochondrial genomes of partial crustacean species.

		**Heavy-strand**		**13 protein-coding genes**		**16S rRNAgene**		**12S rRNA gene**		**22 tRNA genes**		**Control region**		
**Species**	**GenBank accession no.**	**Length (bp)**	**A + T (%)**	**No. of amino acid**	**A + T (%)**	**Length (bp)**	**A + T (%)**	**Length (bp)**	**A + T (%)**	**Length (bp)**	**A + T (%)**	**Length (bp)**	**A + T (%)**	**Reference**
*Charybdis feriata*	KF386147	15,660	70.15	3,716	68.60	1,321	74.26	843	71.89	1,473	71.76	762	78.74	The present study
*Charybdis japonica*	FJ460517	15,738	69.20	3,712	67.80	1,317	74.20	834	70.30	1,458	70.90	863	74.70	21
*Scylla tranquebarica*	NC_012567	15,833	73.80	3,716	72.00	1,339	77.10	869	75.90	1,486	74.40	854	86.50	Unpublished
*Scylla olivacea*	NC_012569	15,723	69.40	3,715	67.30	1,337	74.40	852	72.40	1,482	72.30	778	79.00	Unpublished
*Scylla serrata*	HM590866	15,721	69.22	3,714	69.20	1,337	74.50	839	71.80	1,478	72.26	788	79.10	26
*Scylla paramamosain*	JX457150	15,824	73.04	3,715	70.88	1,340	77.46	869	75.72	1,482	74.56	833	86.67	23
*Portunus trituberculatus*	AB093006	16,026	70.20	3,715	68.80	1,332	73.80	840	70.10	1,468	72.00	1,104	76.30	46
*Callinectes sapidus*	NC_006281	16,263	69.10	3,712	67.00	1,323	71.80	785	70.30	1,463	71.60	1435	78.20	60
*Eriocheir sinensis*	NC_006992	16,354	71.70	3,718	68.90	1,311	77.40	899	76.60	1,473	72.40	896	83.10	61
*Pseudocarcinus gigas*	NC_006891	15,515	70.50	3,734	68.80	1,324	74.90	821	73.80	1,460	73.20	593	80.30	45
*Geothelphusa dehaani*	NC_007379	18,197	74.90	3,711	71.50	1,315	77.10	821	76.40	1,519	75.80	514	87.20	62
*Fenneropenaeus chinensis*	DQ518969	16,004	68.90	3,710	67.50	1,367	72.70	852	69.90	1,501	65.90	997	82.30	25
*Litopenaeus vannamei*	DQ534543	15,989	67.80	3,710	66.10	1,371	71.80	853	69.60	1,493	65.30	998	82.50	25

**Table 3:** The base composition for different regions of mitochondrial genome of *Charybdis feriata* (the genes which are encoded by the L-strand are converted to complementary strand sequences).

	**Base composition (%)**				
**Region**	**A**	**G**	**T**	**C**	**A + T content (%)**
Protein-coding gene					
COI	26.73	15.65	38.20	19.43	64.93
COII	31.97	14.01	34.60	19.42	66.57
ATP8	29.01	9.26	40.12	21.60	69.14
ATP6	27.73	11.36	40.27	20.65	67.99
COIII	26.42	15.55	38.43	19.60	64.85
ND3	29.10	12.15	41.24	17.51	70.34
ND5	31.89	19.16	39.24	9.72	71.12
ND4	29.21	18.73	42.25	9.81	71.46
ND4L	26.07	21.45	42.57	9.90	68.65
ND6	27.02	7.50	45.76	19.72	72.78
Cyt*b*	28.28	13.66	38.06	20.00	66.34
ND1	27.50	19.38	42.08	11.04	69.58
ND2	27.18	8.53	42.46	21.83	69.64
tRNA gene					
tRNA^Leu (UUR)^	34.33	16.42	35.82	13.43	70.15
tRNA^Lys^	26.47	20.59	30.88	22.06	57.35
tRNA^Asp^	41.54	9.23	43.08	6.15	84.62
tRNA^Gly^	42.19	9.38	34.38	14.06	76.56
tRNA^Ala^	35.82	14.93	35.82	13.43	71.64
tRNA^Arg^	32.31	13.85	32.31	21.54	64.62
tRNA^Asn^	41.79	14.93	29.85	13.43	71.64
tRNA^Ser (AGN)^	34.78	14.49	33.33	17.39	68.12
tRNA^Glu^	33.33	13.64	39.39	13.64	72.73
tRNA^His^	31.82	22.73	36.36	9.09	68.18
tRNA^Phe^	40.62	15.62	35.94	7.81	76.56
tRNA^Thr^	38.46	12.31	38.46	10.77	76.92
tRNA^Pro^	34.85	16.67	40.91	7.58	75.76
tRNA^Ser (UCN)^	44.78	13.43	34.33	7.46	79.10
tRNA^Leu (CUN)^	39.13	14.49	37.68	8.70	76.81
tRNA^Val^	28.38	22.97	33.78	14.86	62.16
tRNA^Ile^	35.82	16.42	37.31	10.45	73.13
tRNA^Gln^	31.88	21.74	34.78	11.59	66.67
tRNA^Met^	34.78	13.04	33.33	18.84	68.12
tRNA^Trp^	43.48	11.59	31.88	13.04	75.36
tRNA^Cys^	36.92	15.38	36.92	10.77	73.85
tRNA^Tyr^	35.38	18.46	35.38	10.77	70.77
rRNA gene					
16 S rRNA	38.23	16.58	36.03	9.16	74.26
12 S rRNA	37.25	17.79	34.64	10.32	71.89
Control region	41.99	7.87	36.75	13.39	78.74
Overall of protein-coding genes	28.55	15.25	40.05	16.14	68.60
Overall of tRNA genes	36.25	15.61	35.51	12.63	71.76
Overall of rRNA genes	37.85	17.05	35.49	9.61	73.34
Overall of the genome	34.09	11.25	36.05	18.60	70.15

**Table 4:** Polymorphic sites of 33 haplotypes of complete COI gene of *Charybdis feriata.*, identical.

nucleotide; *N*, number of haplotype in all three localities; AA1, amino acids translated by H1; AA2, amino acids translated by mutated haplotypes.

**Table 5:** Genetic diversity of *Charybdis feriata* from three localities.

**Locality**	**N_H_**	**N**	**h**	**π**
HN	14	21	0.867	0.0012
XM	11	24	0.819	0.0013
ZS	16	25	0.850	0.0011
In total	33	70	0.838	0.0012

*N*_H_, number of haplotypes; *N*, number of individuals; *h*, haplotype diversity; *π*, nucleotide diversity. HN, Hainan locality; XM, Xiamen locality; ZS, Zhoushan locality.

**Table 6:** Analysis of molecular variance (AMOVA) of three localities of *Charybdis feriata*.

**Source of variation**	**df**	**Sum of squares**	**Variance components**	**Percentage of variation**	***F*_ST_**	***P***
Among localities	2	1.898	0.00207 Va	0.23	0.0023	0.293
Within localities	67	60.345	0.90068 Vb	99.77		
Total	69	62.243	0.90274			

**Table 7:** Genetic distance (below diagonal) and fixation index (*F*_ST_) (above diagonal) among three localities of *Charybdis feriata*.

**Locality**	**HN**	**XM**	**ZS**
HN	—	−0.0040	−0.0002
XM	0.001	—	0.0098
ZS	0.001	0.001	—

HN, Hainan locality; XM, Xiamen locality; ZS, Zhoushan locality.

**Table 8:** Neutrality tests results of *Charybdis feriata*.

	**Ewens-Watterson**		**Chakraborty**			
**Locality**	**F_O_**	**F_E_**	**N_O_**	**N_E_**	**Tajima’s *D***	**Fu’s *Fs***
HN	0.17	0.10	14	5.09*	−2.46^**^	−12.19**
XM	0.22	0.15	11	5.53	−2.16^**^	−5.35**
ZS	0.18	0.09	16	5.14**	−2.40^**^	−15.73**
Mean	0.19	0.11	13.67	5.25*	−2.34^**^	−11.09**

*F*_O_, observed *F* value; *F*_E_, expected *F* value; *N*_O_, number of observed alleles; *N*_E_, number of expected alleles; HN, Hainan locality; XM, Xiamen locality; ZS, Zhoushan locality; ^*^, P < 0.05; ^**^, *P *< 0.01.

**Table 9:** Mismatch distributions analysis of *Charybdis feriata*.

**Locality**	**τ**	**θ_0_**	**θ_1_**	**SSD (*P*_SSD_)**	**Rag (*P*_Rag_)**
HN	1.645	0.000	99999.000	0.013 (0.254)	0.109 (0.172)
XM	1.438	0.000	99999.000	0.012 (0.229)	0.106 (0.190)
ZS	1.617	0.000	99999.000	0.008 (0.332)	0.095 (0.194)
Mean	1.566	0.000	99999.000	0.011 (0.272)	0.103 (0.186)

*τ*, units of mutational time; *θ*_0_, *θ* before population growth; *θ*_1_, *θ* after population growth; SSD, sum of the square deviations between the observed and expected mismatch; *P*_SSD_, the probability of SSD; Rag, raggedness index; *P*_Rag_, the probability of raggedness; HN, Hainan locality; XM, Xiamen locality; ZS, Zhoushan locality.
