# Supplementary material for: First mitochondrial genome for the red crab (Charybdis feriata) with implication of phylogenomics and population genetics
Source: Sci Rep. 2015 Jul 30;5:11524. doi: 10.1038/srep11524 (PMC4520191; doi:10.1038/srep11524)
Supplement: Supplementary Information [file srep11524-s1.doc]

**First mitochondrial genome for the red crab (*Charybdis feriata*) with implication of phylogenomics and population genetics**

Hongyu Ma ^1, 2^, Chunyan Ma ^1, 2^, Chenhong Li ^3^, Jianxue Lu ^1, 2^, Xiong Zou ^1, 2^, Yangyang Gong ^1, 2^, Wei Wang ^1, 2^, Wei Chen ^1, 2^, Lingbo Ma ^1, 2^, Lianjun Xia ^1, 2^

**Supplementary Table 1.** **Primers used for amplification of mitochondrial genome of *Charybdis feriata*.**

| **Primer Name** | **Sequence (5’ – 3’)** | **Annealing temperature (℃)** |
| --- | --- | --- |
| J | TAAACTAAAAGCCTTCAAAGCTTAA  GAAGGAGGAAGAAGTCAAAATCTT | 53 |
| COI-A2-2 | CAACGATGATTCTTTTCTACAAA  TGTAGTGTAAGCGTCAGGATAA | 53 |
| COI-A2-1 | GGTGTMGGYACWGGWTGAACTG  TGTAGTGTAAGCGTCAGGATAA | 55 |
| COI-1-A2 | GGCTTTCACTAAATCCTAAATGAAT  AATYARYTCAATAGTTTGATGTTC | 52 |
| COI-1-A3 | GGCTTTCACTAAATCCTAAATGAAT  GACCTACACTTWCTTTCAGTCATC | 52 |
| A3-COI3 | GGGATTATTCTACGGCCAGTG  AGATGTTATTGAAGGGCCAGTATAC | 59 |
| A3-COI3-2 | ATAGTACGTCCGCCTACTTCT  GTGGTTGATTCAGCCTCTAAT | 58 |
| A | TTTGCCCATCTTGTACCTCAA  TTCTCGAGTAACATCACGCCA | 56 |
| COI-3 | AYTRGCHGCYAATATAATTGCAGG  CYTTCTGATTGGAAGTCAGATATAC | 56 |
| COI3-4 | TTATCCTCTGGAGCAACTGT  GACCCACTCATAATAAAGACCTAC | 57 |
| COI-4 | ATCTGACTTCCAATCAGAARGTCT  AATATTGCATTGAAGCTGCAAAAG | 54 |
| COI4-C2 | TTTCGTTGTAAAGCTGAAGG  TTCAATGTGTGGGTTAGGAGTA | 54 |
| C-2 | AGCTGAAGGCTTACCTTCTA  TTATTTCWTGTYTRACAATRTTTAT | 50 |
| D-2 | GAATAACTACCCCAGCACAT  MTTATTTTGYTTRGCTAATATAGT | 51 |
| E-1 | TTAAAGAAGGAGGCCTAGCCAT  TRGTMCGRAGSCATGGAAATGA | 57 |
| E-G | TACCTGAACAATTTTCCATTCA  AAATTAAGATGGTTTGAACAAGTAA | 53 |
| G | AGGWAWWATAAAYTTTAACATTCTA  RATATGDGCWACTCTAGARAAT | 50 |
| G-16S | GTTGATATACCTCTACCGTCAAACA  TTATTTGGTTGGGGCGACAG | 57 |
| G-16-2 | ATCCCTTAGTTACCCCTGCTC  CATGAAGAGTTATGCCTTATGATGT | 54 |
| 16-12 | TTCTTTTATAGCTGCTGCACTAT  GAAGGAGGATTTGATTGTAAGA | 54 |
| B | CTATCCAGACTCACTTTCCAG  ACCTGTTTTTGAATCGATAAA | 51 |
| P | TGCACCTTGATCTAATATATTTAAC  GAAAGGTCCCTGTTAGTAATGTA | 53 |
| R | TTATTCACTCCTAAACTTTTAACACA  GGATGTCAATTATGCGGGTA | 55 |
| N | TAATGGGCTCATACCCCGTA  TTTGTAGAAAAGAATCATCGTTGC | 54 |
| O | AYYGAAGGYYTAWCWTGACCKCAAG  CTGGTTGACCTAGTTCGGCTCGAA | 60 |
